# Predicting Food Effect On Oral Drug Absorption For Solubility-Epithelial Membrane Permeation-Limited Cases With Bile Micelle Solubilization

**DOI:** 10.1007/s11095-025-03947-8

**Published:** 2025-10-14

**Authors:** Yuji Higashiguchi, Shiori Ishida, Samuel Lee, Balint Sinko, Karl Box, Kiyohiko Sugano

**Affiliations:** 1 Molecular Pharmaceutics Lab., College of Pharmaceutical Sciences, Ritsumeikan University, 1-1-1, Noji-Higashi, Kusatsu, Shiga 525-8577 Japan; 2 Pion Inc. (UK) Ltd. Forest Row Business Park, Station Road, East Sussex, RH18 5DW UK

**Keywords:** Dissolution, Food effect, Free fraction, Oral absorption, Permeation, Unstirred water layer

## Abstract

**Purpose:**

The purpose of this study was to investigate the theoretical and *in vitro* experimental prediction of food effects on oral drug absorption, focusing on solubility-epithelial membrane permeation-limited cases (SL-E).

**Method:**

Bosentan, fidaxomicin, pranlukast, and rifaximin were employed as model SL-E drugs. Celecoxib and danazol were employed as solubility-unstirred water layer permeation-limited cases (SL-U) for comparison. Theoretical predictions of food effects were based on the rate-limiting steps of the fraction of a dose absorbed (*Fa*) (FaRLS) (Fa rate-limiting step). μFLUX was used as a dissolution-permeation flux (*J*_*μFLUX*_) experiment. Fasted and fed state simulated intestinal fluids (FaSSIF and FeSSIF, respectively) were employed as the donor solution.

**Results:**

For all SL-E drugs, the food effect on *Fa* was theoretically predicted to be 1.2, irrespective of bile micelle solubilization (FaSSIF/FeSSIF: bosentan (2.1), fidaxomicin (2.3), pranlukast (9.1), and rifaximin (3.5)). Theoretically, an increase in solubility by bile micelles is counterbalanced by a decrease in effective permeability (*P*_*eff*_) due to a decrease in the free fraction (*P*_*eff*_ is defined based on unbound + bound drug concentration (*C*_*D*_)). This prediction was consistent with the clinical data (fed/fasted AUC ratio: 1.1, 1.0, 1.3, and 1.6, respectively). In μFlux, even though *C*_*D*_ was markedly higher in FeSSIF than in FaSSIF (1.9, 3.1, 20, and 3.3-fold, respectively), *J*_*μFLUX*_ was less enhanced (0.91, 0.81, 2.4, and 0.81-fold, respectively). For the SL-U drugs, as theoretically expected, *J*_*μFLUX*_ was increased as *C*_*D*_ was increased, which was consistent with the clinical data.

**Conclusion:**

FaRLS appropriately predicted the food effect for the SL-E drugs. The mechanism was experimentally confirmed by μFlux.

**Supplementary Information:**

The online version contains supplementary material available at 10.1007/s11095-025-03947-8.

## Introduction

Predicting the food effect on the oral absorption of drugs remains challenging [1]. The gastrointestinal (GI) conditions in the fed state differ from those in the fasted state [2, 3]. For example, the concentration of bile micelles increases about fivefold on average in the fed state compared to the fasted state [4–8]. Bile micelles affect the dissolution, supersaturation, and precipitation profiles of a drug (hereinafter referred to as the dissolution profile) [4, 9, 10]. Furthermore, bile micelles reduce the effective intestinal permeation of most drugs [11–25]. Several theoretical and empirical frameworks have been developed for predicting food effects [26–32]. In addition, various *in vitro* dissolution-permeation flux experiments have also been applied to predict food effects [14, 33–36]. In real-world drug discovery and development, theoretical and experimental methods are used together to provide a comprehensive approach for predicting food effects [35].

In theory, the effect of bile micelles on the fraction of a dose absorbed (*Fa*) depends on the rate-limiting step (RLS) (FaRLS) (see theory section) (Table I) [26, 37]. FaRLS categorizes the RLS of oral drug absorption into 5 classes: dissolution rate limited (DRL), epithelial membrane (EPM) permeation limited (PL-E), unstirred water layer (UWL) permeation limited (PL-U), solubility—EPM permeation limited (SL-E), and solubility—UWL permeation limited (SL-U).

**Table I:** FaRLS and Food Effect By Bile Micelles ^a^

Rate-limiting step	Abbreviation	Criteria ^d^	Food effect [*Fa* ratio (fed/fasted)]
Dissolution	DRL	*Dn* < *Pn*/*Do*	Positive (> 1)
EPM^b^ permeation	PL-E	*Dn* > *Pn*/*Do*, *Do* < 1, *P*_*ep*_*’* < *P*_*UWL*_	Negative (< 1)
UWL^c^ permeation	PL-U	*Dn* > *Pn*/*Do*, *Do* < 1, *P*_*ep*_*’* > *P*_*UWL*_	None (≈ 1)
Solubility – EPM permeation	SL-E	*Dn* > *Pn*/*Do*, *Do* > 1, *P*_*ep*_*’* < *P*_*UWL*_	Slightly positive (≈ 1.2)
Solubility – UWL permeation	SL-U	*Dn* > *Pn*/*Do*, *Do* > 1, *P*_*ep*_*’* > *P*_*UWL*_	Positive (> 1)

^a^ Attributed to bile micelle solubilization (binding).  ^b^*EPM* epithelial membrane. ^c^*UWL* unstirred water layer.  ^d^See theory section

**Fig. 1:**
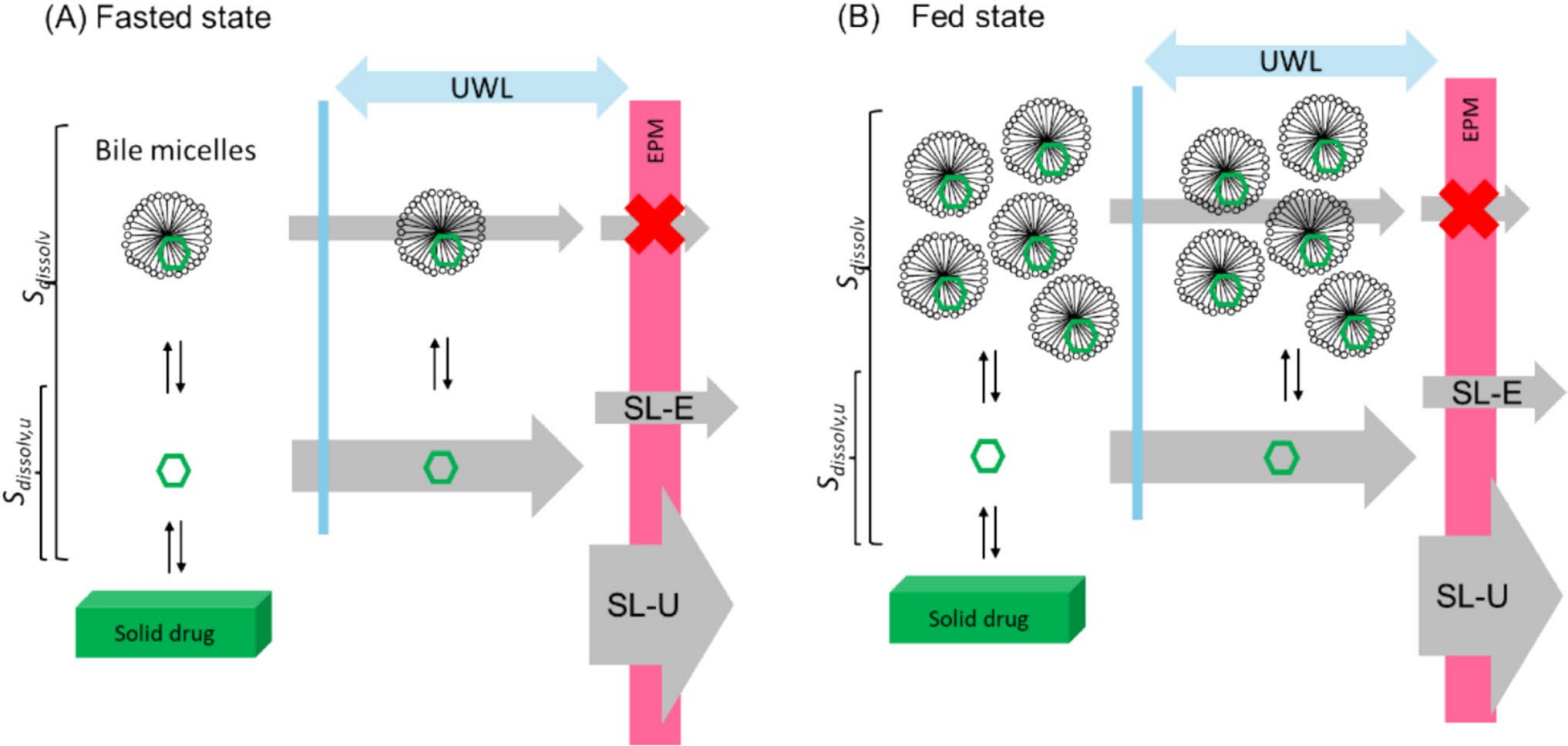
Bile micelle solubilization and permeation flux (dimension: amount/area/time). (**A**) Fasted state, (**B**) fed state. The width of the grey arrows indicates the permeability of a drug across the unstirred water layer (UWL) and epithelial membrane (EPM) (dimension: length/time). The “effective” permeability of a drug (*P*_*eff*_) is defined based on the total drug concentration dissolved in the intestinal fluid (bile micelle-bound + unbound drugs).

In bile micelle media, drug molecules exist as bile micelle-bound and unbound species in rapid equilibrium (Fig. 1). The solubility of a drug (*S*_*dissolv*_) is defined as the sum of the concentrations of these species in equilibrium with an undissolved solid-state drug. When the bile micelle concentration increases in the fed state, *S*_*dissolv*_ increases. However, the concentration of unbound (free) drug remains the same (*S*_*dissolv,u*_). Both bile micelle-bound and unbound drug molecules can permeate UWL adjacent to EPM, whereas only unbound (free) drug molecules can permeate EPM (free drug theory) [38–42]. Therefore, in the case of SL-E drugs, *Fa* is not increased even when *S*_*dissolv*_ is increased by bile micelles in the fed state because the free drug concentration remains the same (= *S*_*dissolv,u*_). In other words, an increase in *S*_*dissolv*_ by bile micelles is counterbalanced by a decrease in the “effective” permeability (*P*_*eff*_). The important point here is that *P*_*eff*_ and *S*_*dissolv*_ are formally defined based on the total concentration of a drug dissolved in intestinal fluid (bile micelle-bound + unbound drugs). Therefore, *Fa* and flux (*J*) are in proportion to *S*_*dissolv*_ × *P*_*eff*_ in SL-E and SL-U cases (see the theory section) [11–13, 15–24, 41].

Food effect prediction by FaRLS has been systematically evaluated for PL-E [24, 43, 44] and SL-U drugs [26, 45], as well as hydrophilic SL-E drugs that do not interact with bile micelles [26]. However, this prediction scheme has not yet been validated by multiple SL-E drugs that undergo bile micelle solubilization. Only one example has been reported in the literature (pranlukast) [26].

**Fig. 2:**
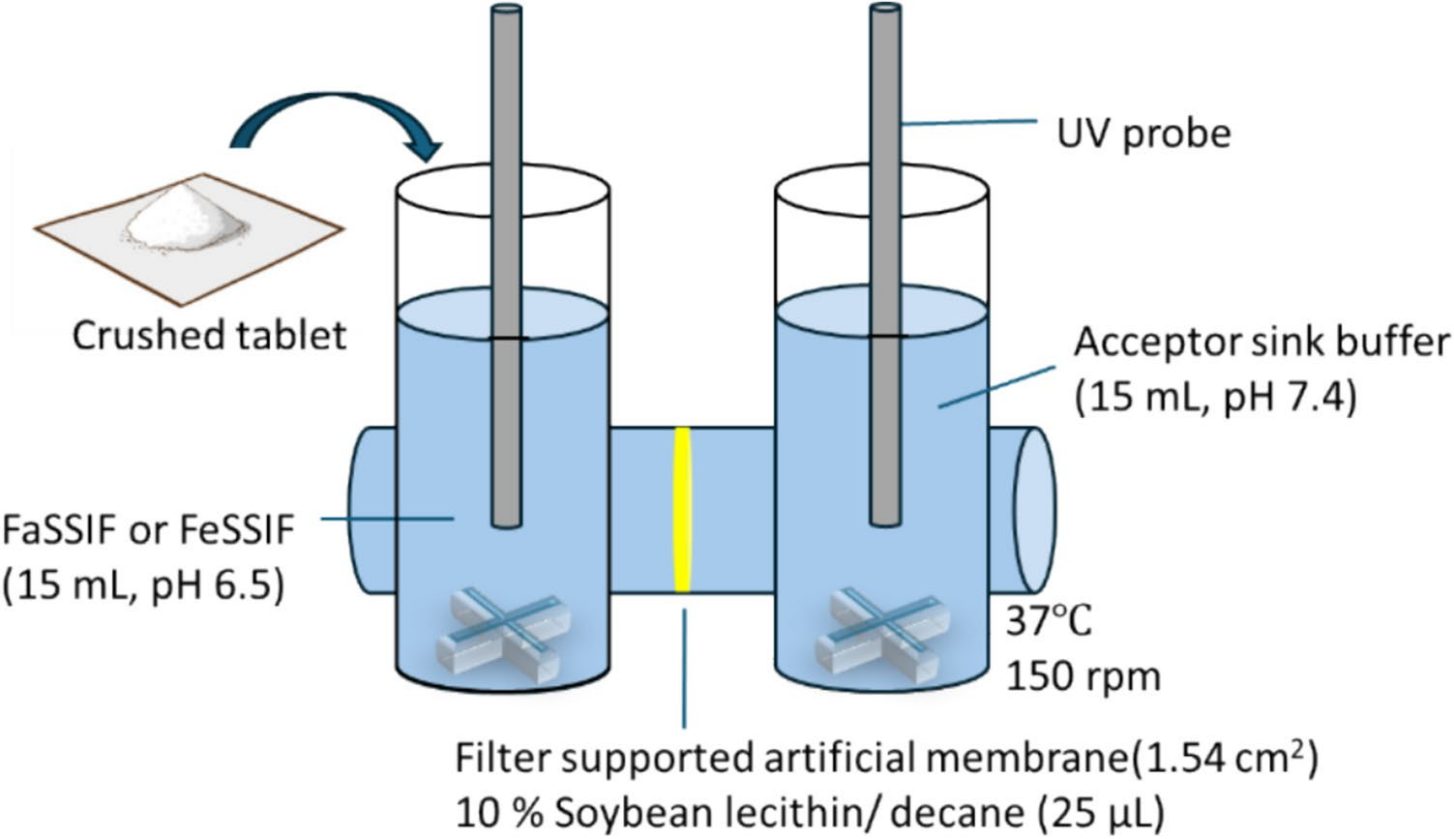
μFlux experiment.

Various *in vitro* dissolution-permeation flux experiments, such as the dissolution/permeation system, μFlux, and macroFlux, have been investigated for food effect prediction (Fig. 2) [14, 33–36]. In these studies, SL-U drugs were mainly used as model drugs. However, little is known about their applicability for SL-E drugs. Previously, Kataoka *et al.* showed that the food effect on *Fa* of pranlukast can be appropriately predicted by the dissolution/permeation system using Caco-2 [14]. However, no other case of SL-E drugs has been reported.

**Fig. 3:**
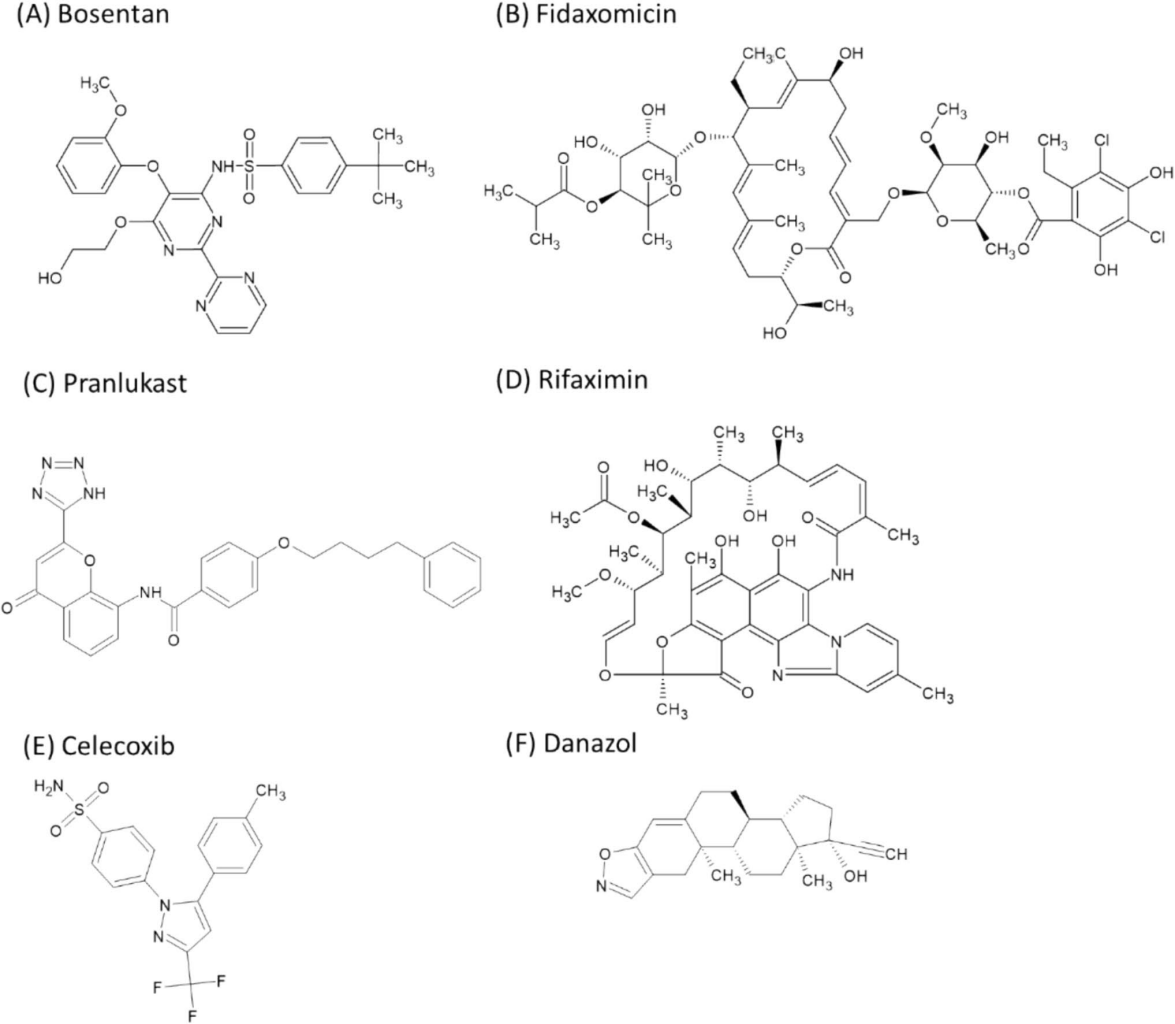
Chemical structures of model drugs.

The purpose of the present study was twofold: firstly, to validate the theoretical food-effect prediction for SL-E drugs by increasing the number of model drugs, and secondly, to confirm the mechanism through dissolution-permeation flux experimentation. In this study, bosentan, fidaxomicin, pranlukast, and rifaximin were employed as model SL-E drugs (Fig. 3). For comparison, celecoxib and danazol were employed as model SL-U drugs [36]. The physicochemical properties of these drugs are shown in Table II. Bosentan, fidaxomicin, and rifaximin are beyond-rule-of-five drugs (bRO5). bRO5 drugs have attracted a lot of interest in recent drug discovery and development [46, 47]. The bioavailability of these model drugs is incomplete in humans [48–51]. In this study, the food effect was predicted using the GUT framework, a simple theoretical model for oral drug absorption. This framework is completely transparent and publicly available [26]. FaRLS is theoretically based on this framework. In addition, μFLUX was used to further understand the mechanism of the food effect by bile micelles. The fasted and fed state simulated intestinal fluid (FaSSIF and FeSSIF, respectively) [4, 5] were used as the donor solution in μFLUX.

**Table II:** Physicochemical Properties and Caco-2 Permeability of Model Drugs

Drug	MW	p*K*_*a*_^*a*^	log*P*_*oct*_^b^	log*D*_*oct,pH 6.5*_^*c*^	Solubility (μg/mL)^d^			Caco-2 *P*_*app*_ (10^–6^ cm/s)
					Blank FaSSIF	FaSSIF	FeSSIF	
SL-E								
Bosentan	552	4.87 (A)	3.8	2.2	30 ± 1	41 ± 1	87 ± 1	1 [52]
Fidaxomicin	1058	5.15 (A) 9.09 (A)	3.7	2.3	46 ± 1	77 ± 1	178 ± 2	< 0.092 [53]
Pranlukast	482	-^e^	4.2^f^	-^e^	3	88	800	0.08 [54]
Rifaximin	786	< 2.0 (A) 8.29 (B)	2.6	2.6	1.8 ± 0	3.4 ± 0.0	12 ± 0	1 [55]
SL-U								
Celecoxib	381	-	3.5	3.5	3.2	46.2	103	18 [56]
Danazol	337	-	4.5	4.5	0.2	5–18	18–47	14 [13], 29.2 [19]

a Measured in this study (ionic strength (*I*) = 0.168 mol/L, 37°C except for pranlukast (25°C)). b Log*P*_*oct*_ obtained from the literature (bosentan [48], fidaxomicin [49, 57], rifaximin [58, 59], celecoxib [60], danazol [61]). c Calculated from log*P*_*oct*_ and p*K*_*a*_ using the Henderson-Hasselbalch equation. d Measured in this study for bosentan, fidaxomicin, and rifaximin. pH 6.5 for all conditions. Mean ± S.D., N = 3. Literature data for pranlukast [26], celecoxib [60], and danazol [9, 62]. e Pranlukast has an acidic functional group (tetrazole). However, it was difficult to determine the p*K*_*a*_ value due to its extremely poor solubility. f *In silico* calculated value (Advanced Chemistry Development (ACD/Labs) Software V8.14)

## Theoretical Framework

### *Fa* Equation

In this study, the food effect on *Fa* was predicted based on the unified theoretical framework for GI drug absorption (the GUT framework) [26]. Previously, the GUT framework has been explained in detail in the literature [45, 63, 64]. Therefore, it is briefly explained in the following. The GUT framework can also be applied to μFLUX (*Fa* in μFLUX is the fraction absorbed into the acceptor compartment).

In the case when the gastric dissolution process of a drug is negligible, *Fa* can be described by the *Fa* equation [65].

1 $$Fa=1-exp\left(-\frac1{\frac1{Dn}+\frac{Do}{Pn}}\right),if\;Do<1,set\;Do=1$$

where *Dn*, *Pn*, and *Do* are the dimensionless parameters representing the dissolution process (dissolution number, *Dn*), the permeation process (permeation number, *Pn*), and the ratio of a dose strength (*Dose*) to the solubilization capacity (dose number, *Do*) in the small intestine [66].

2 $$Dn={k}_{diss}{T}_{SI}$$

3 $$Pn={k}_{perm}{T}_{SI}$$

4 $$Do=\frac{Dose}{{S}_{dissolv}{V}_{SI}}$$

where *k*_*diss*_ is the dissolution rate constant, *k*_*perm*_ is the first-order permeation rate constant, *T*_*SI*_ is the small intestinal transit time (3.5 h for humans), *V*_*SI*_ is the small intestinal fluid volume (130 mL and 156 mL in fasted and fed humans, and 15 mL for μFLUX) [63, 67–71], and *S*_*dissolv*_ is the equilibrium solubility of a drug in the human small intestinal fluid or the donor medium in μFLUX.

When *Dn* >  > *Pn*/*Do*, the oral absorption of a drug becomes solubility-limited (SL). In this case, the *Fa* equation (Eq. (1)) can be approximated to,

5 $$Fa= 1-exp\left(-\frac{Pn}{Do}\right)$$

When *Fa* < 0.7, it can be further approximated to [66],

6 $$Fa=\frac{Pn}{Do}$$

### Effective Intestinal Permeability

The permeation rate constant (*k*_*perm*_) is related to the effective intestinal permeability (*P*_*eff*_). *P*_*eff*_ is defined based on the total concentration of a drug dissolved in intestinal fluid (bile micelle-bound + unbound drugs).

7 $${k}_{perm}=\frac{SA}{{V}_{SI}}{P}_{eff}=\frac{2DF}{{R}_{SI}}{P}_{eff}$$

where *SA*, *R*_*SI*_, and *DF* are the smooth surface area, the radius, and the degree of flatness of the small intestine, respectively (for a cylindrical tube (*DF* = 1) with a length (*L*), *SA*/*V*_*SI*_ = 2π*R*_*SI*_*L*/π*R*_*SI*_^2^*L* = 2/*R*_*SI*_). *R*_*SI*_ and *DF* are 1.5 cm and 1.7 in humans. *SA* = 1.54 cm^2^ in μFLUX. *Pn* is originally introduced by Oh *et al.* as the absorption number (*An* = *P*_*eff*_/*R*_*SI*_ × *T*_*SI*_ = 1/2 × *Pn*) [66]. The number “1/2” originates from the cylindrical tube shape of the small intestine. Therefore, *An* cannot be applied to μFLUX. The use of *Pn* is theoretically more consistent and is therefore employed in the GUT framework.

As mentioned in the introduction, both bile micelle-bound and unbound drug molecules can permeate UWL adjacent to EPM. However, only unbound drug molecules can permeate EPM (free drug theory) [26]. Considering the surface expansion by the plicae circulares (*PE*) (3 for humans, 1 for μFLUX) and the villi (*VE*) (10 for humans, 1 for μFLUX), *P*_*eff*_ can be described as [40],

8 $${P}_{eff}=\frac{PE}{\frac{1}{{P}_{UWL}}+\frac{1}{{{P}_{ep}}{\prime}}}=\frac{PE}{\frac{1}{{P}_{UWL}}+\frac{1}{VE{f}_{u}{P}_{ep,u}}}$$

where *P*_*UWL*_ is the UWL permeability, *f*_*u*_ is the unbound fraction, *P*_*ep,u*_ is the permeability of the epithelial membrane (*in vivo*) or the artificial membrane (μFlux) for unbound drug molecules. This equation is called the *P*_*eff*_ equation.

#### Unbound Fraction in Biorelevant Media

The *f*_*u*_ value can be calculated as the ratio of solubility in the absence and presence of bile micelles (*S*_*dissolv,u*_ and *S*_*dissolv*_, respectively) (Fig. 1).

9 $${f}_{u}= \frac{{S}_{dissolv,u}}{{S}_{dissolv}}$$

In the present study, *S*_*dissolv,u*_ corresponds to the equilibrium solubility in the blank FaSSIF (no bile micelles), and *S*_*dissolv*_ corresponds to that in FaSSIF or FeSSIF (both pH 6.5).

#### Epithelial Membrane Permeability

*P*_*ep,u*_ can be assumed to be the same as the *in vitro* apparent permeability (*P*_*app*_), such as Caco-2 *P*_*app*_ (*P*_*ep,u*_ = *P*_*app*_) if *P*_*app*_ is not limited by UWL (*P*_*app*_ < 20 × 10^–6^ cm/s with typical Caco-2 assay setting) and not interfered with poor recovery (see discussion). In other cases, *P*_*ep,u*_ can be roughly estimated from the physicochemical properties of a drug (this estimation is sufficient for SL-U drugs because UWL permeation is the rate-limiting step) [63].

#### Unstirred Water Layer Permeability

*P*_*UWL*_ can be calculated as,

10 $${P}_{UWL}= \frac{{{f}_{u}D}_{mono}+{\left(1-{f}_{u}\right)D}_{bm}}{{h}_{UWL}}+{P}_{WC}$$

where *D*_*mono*_ and *D*_*bm*_ are the diffusion coefficients of unbound drugs and bile micelle-bound drugs, respectively, *h*_*UWL*_ is the effective thickness of the UWL, and *P*_*WC*_ is the UWL permeation by water conveyance (only for *in vivo*) (*D*_*bm*_ = 0.13 and 1.05 × 10^–6^ cm^2^/sec for FaSSIF and FeSSIF, respectively (in the absence of mucus)) [39]. In μFLUX, *h*_*UWL*_ was estimated to be 164 μm at the stirring rate of 150 rpm (pION in-house data).

### Food Effect Prediction For Solubility-Epithelial Membrane Permeation Limited Cases

In the case of SL-E drugs (*P*_*ep*_*’* <  < *P*_*UWL*_), from Eqs. (3) to (8),

11 $$Fa=\left(\frac{{S}_{dissolv,u}}{{f}_{u}}\frac{{V}_{SI}}{Dose}\right)\times \left(\frac{2DF}{{R}_{SI}}PE\bullet VE\bullet {f}_{u}\bullet {P}_{ep,u}{\bullet T}_{SI}\right)=\frac{{S}_{dissolv,u}{V}_{SI}}{Dose}\frac{2DF}{{R}_{SI}}PE\bullet VE\bullet {P}_{ep,u}{\bullet T}_{SI}$$

In this equation, *f*_*u*_ is cancelled out. Therefore, in the case of SL-E, it is theoretically suggested that bile micelle solubilization does not affect *Fa*. In other words, when *S*_*dissolv*_ is increased by bile micelles (*S*_*dissolv*_ = *S*_*dissolv,u*_/*f*_*u*_), *P*_*eff*_ is decreased to the same extent (*P*_*eff*_ ∝ *f*_*u*_*P*_*ep,u*_). Therefore, in the case of SL-E drugs,

12 $$Fa\propto {S}_{dissolv}\times {P}_{eff}=\frac{{S}_{dissolv,u}}{{f}_{u}}{f}_{u}\bullet {P}_{ep,u}={S}_{dissolv,u}\times {P}_{ep,u}$$

When assuming that the parameters other than *V*_*SI*_ are the same between the fed and fasted states, the fed/fasted ratio of *Fa* (*Fa*_*fed*_/*Fa*_*fasted*_) equals that of *V*_*SI*_.

13 $$\frac{{Fa}_{fed}}{{Fa}_{fasted}}=\frac{{V}_{SI,fed}}{{V}_{SI,fasted}}$$

where the additional subscript indicates the fed or fasted state.

The fed/fasted ratio of *V*_*SI*_ has been estimated to be about 1.2 in humans, based on the clinical AUC ratio of several hydrophilic un-ionizable SL-E drugs for which *Fa* would not be affected by bile micelles and pH [26]. Therefore, the food effect on *Fa* for SL-E drugs is predicted to be only slightly positive (1.2-fold) in humans, even when *S*_*dissolv*_ is increased by bile micelles in the fed state.

Similarly, in μFLUX, the flux value (*J*_*μFLUX*_, dimension: amount/area/time) is predicted to be not affected by bile micelles.

14 $${J}_{\mu FLUX}={S}_{dissolv}{P}_{eff}=\frac{{S}_{dissolv,u}}{{f}_{u}}{f}_{u}\bullet {P}_{ep,u}={S}_{dissolv,u}\bullet {P}_{ep,u}$$

where *P*_*eff*_ is the effective permeability in μFLUX. Therefore, in the case of SL-E drugs, the fed/fasted ratio of *J*_*μFLUX*_ is predicted to become 1.0 (*V*_*IS*_ is the same for both conditions in μFLUX).

### Food Effect Prediction For Solubility-Unstirred Water Layer Permeation Limited Cases

In the case of SL-U drugs (*P*_*UWL*_ <  < *P*_*ep*_*’*),

15 $$Fa=\frac{{S}_{dissolv}{V}_{SI}}{Dose}\times \frac{2DF}{{R}_{SI}}PE\bullet VE\bullet {P}_{UWL}{\bullet T}_{SI}$$

Therefore,

16 $$\frac{{Fa}_{fed}}{{Fa}_{fasted}}=\frac{{S}_{dissolv,fed}{P}_{UWL,fed}{V}_{SI,fed}}{{S}_{dissolv,fasted}{{P}_{UWL,fasted}V}_{SI,fasted}}$$

For SL-U drugs, the food effect is predicted to be positive because an increase in *S*_*dissolv*_ is not cancelled out by a decrease in *P*_*UWL*_ (See ref. [26] for details).

### Rate-Limiting Step of *Fa*

The rate-limiting step of *Fa* (FaRLS) can be diagnosed by the *Fa* equation (Eq. (1)) and the *P*_*eff*_ equation (Eq. (8)) (Table I) [26, 39]. Based on the *Fa* equation, oral drug absorption can be categorized into dissolution rate limited (DRL) (*Dn* < *Pn*/*Do*), permeability limited (PL) (*Dn* > *Pn*/*Do*, *Do* < 1), and solubility-permeability limited (SL) (*Dn* > *Pn*/*Do*, *Do* > 1) [37, 39]. The *P*_*eff*_ equation further categorizes PL into PL-E and PL-U, and SL into SL-E and SL-U.

### Food Effect Prediction Using a Simple Empirical *P*_*eff*_ Prediction

A simple empirical correlation between *P*_*eff*_ and *P*_*app*_ has often been employed to predict *P*_*eff*_ in physiologically based biopharmaceutics modeling (PBBM) using a commercial software program, such as GastroPlus and simCYP.

17 $${logP}_{eff}=alog{P}_{app}+b$$

where *a* and *b* are empirical coefficients determined from a log*P*_*eff*_ – log*P*_*app*_ correlation. For Eq. (17), *P*_*app*_ is typically measured by Caco-2 in the absence of bile micelles. This equation ignores the free drug theory and UWL, implicitly suggesting that the *P*_*eff*_ values in the fasted and fed states are the same. In this case, when assuming that the parameters other than *S*_*dissolv*_ are the same between the fed and fasted states for both SL-E and SL-U cases,

18 $$\frac{{Fa}_{fed}}{{Fa}_{fasted}}\approx \frac{{S}_{dissolv,fed}}{{S}_{dissolv,fasted}}$$

## Materials and Methods

### Materials

Bosentan, fidaxomicin, pranlukast, celecoxib, and rifaximin were purchased from Tokyo Chemical Industry Co., Ltd (Tokyo, Japan). Danazol was purchased from Sigma-Aldrich Co. (MO, USA). Sodium taurocholic acid (TC), sodium chloride, sodium dihydrogen phosphate dihydrate, 6 N HCl, and 8 N NaOH were purchased from FUJIFILM Wako Pure Chemical Corporation (Osaka, Japan). Egg yolk lecithin (EL) was purchased from Kewpie Corporation (Tokyo, Japan). Soybean lecithin was provided by Tsuji Oil Mills Co., Ltd (Mie, Japan) (SLP-White, phosphatidylcholine (24—32%), phosphatidylethanolamine (20—28%), phosphatidylinositol (12—20%), phosphatidic acid (8—15%), and lysophosphatidylcholines (1—5%) (based on the product information provided by the manufacturer)). The acceptor sink buffer was provided by pION Inc. (MA, USA).

### Methods

#### p*K*_*a*_ Measurement

The p*K*_*a*_ measurements were performed using a SiriusT3 automatic titrator, with a spectrometric (UV-metric) or titration (pH-metric) method. The UV-metric method used a fiber optic dip probe, a deuterium lamp UV light source, and a photodiode array detector to capture the absorption spectra of a sample solution over the course of three titrations, across the pH range 2.0–12.0. Due to low aqueous solubility, the drugs were titrated in a water–methanol co-solvent medium, over a concentration range of 31–20 µM, where the co-solvent percentage was altered between titrations. Titrations were carried out under a controlled temperature of 37 ± 0.2°C.

SiriusT3 software v2.0 was used to apply principal component analysis to the spectral data to resolve the molar absorptivity spectra of each sample species and determine the p_s_*K*_*a*_ values of the compounds in the co-solvent. The p*K*_*a*_ values under aqueous conditions were calculated from the co-solvent data using the Yasuda-Shedlovsky extrapolation method.

Additional potentiometric titrations were performed for each drug, which confirmed that no additional non-UV active p*K*_*a*_s were present within the titration pH range.

#### Equilibrium Solubility Measurement

A shake-flask method was used for equilibrium solubility measurement. Each drug (10 mg) was added to the 15 mL test tube. Blank FaSSIF (28.7 mM phosphate buffer, pH 6.5 adjusted by NaOH), FaSSIF (3 mM TC, 0.75 mM EL in blank FaSSIF), or FeSSIF (15 mM TC, 3.75 mM EL in blank FaSSIF) was added. The test tube was placed horizontally in a reciprocal shaker. After shaking for 72 h at 37°C (at 90 rpm, motion width: 2.5 cm), the sample was filtered (hydrophilic PVDF, φ = 4.0 mm, pore size: 0.22 µm, Merck). The first few droplets were discarded to avoid filter adsorption. The concentration of each drug was measured by UV absorbance. The wavelength for each drug is shown in Supplemental Information Table S1. After measuring the final pH (9615S-10D Standard ToupH electrode (HORIBA Advanced Techno Co., Ltd., Kyoto, Japan)), the residual particles were collected by vacuum filtration and analyzed by powder X-ray diffraction (PXRD) (Supplemental Information Figure S1).

#### μFLUX Experiment

The μFLUX apparatus (pION Inc., MA, USA) (Fig. 2) was used for dissolution-permeation flux experiments. The membrane filter (1.54 cm^2^) was coated with 25 μL of a 10% soybean lecithin-decane solution [72]. FaSSIF or FeSSIF (15 mL) was added to the donor chamber. The acceptor sink buffer (volume (*V*_*A*_) = 15 mL) was added to the acceptor chamber. The temperature was maintained at 37°C. The stirring rotation speed was set to 150 rpm. Each drug formulation (1/10 of the clinical dose) was added after being gently crushed by mortar and pestle. The drug concentrations in the donor (*C*_*D*_) and acceptor (*C*_*A*_) solutions were monitored using a UV probe for 2 h, except for pranlukast (4 h) (2 mm (donor) and 20 mm (acceptor) apertures). The wavelength for each drug is shown in Supplemental Information Table S2. The drug concentration in the donor chamber was calculated after correcting for turbidity by subtracting the absorbance at 500 nm, except for rifaximin (600 nm). The final drug concentration in the donor chamber (*C*_*D, final*_) was measured after filtration as described above.

The flux value (*J*_*μFLUX*_) was calculated from the slope of the drug concentration–time curve in the acceptor solution in the last 30 min.

19 $${J}_{\mu FLUX}=\frac{\Delta {C}_{A}}{\Delta T}\frac{{V}_{A}}{SA}$$

where *SA* is the membrane surface area (1.54 cm^2^). The effective permeability in μFLUX (*P*_*eff, μFLUX*_) value was calculated from *J*_*μFLUX*_ and *C*_*D, final*_.

20 $${P}_{eff,\mu FLUX}=\frac{{J}_{\mu FLUX}}{{C}_{D,final}}$$

## Results

### Physicochemical Properties of Model Drugs

Bosentan, fidaxomicin, and pranlukast are low-solubility weak acids (Table II). The p*K*_*a*_ value of bosentan was 4.87. For fidaxomicin, two acidic p*K*_*a*_ values were observed (5.15 and 9.09). Pranlukast has an acidic functional group (tetrazole). However, it was difficult to determine the p*K*_*a*_ value due to its extremely poor solubility in this study. In the case of rifaximin, one basic p*K*_*a*_ value of 8.29 was observed in the present study. Rifaximin also has one acidic p*K*_*a*_ below 2.0, which is related to the 4-hydroxyl group (the p*K*_*a*_ value of a structurally similar drug, rifampicin, was reported to be 1.7 [73]). Therefore, rifaximin is a zwitterionic drug. The solubility of a zwitterionic drug remains almost constant in the pH range between its two p*K*_*a*_*s*. Celecoxib and danazol have no ionizable groups in the physiological pH range. All these drugs are formulated in their free form (Table III).

**Table III:** Drug Product Used for μFLUX Experiment

Drug	Product	Manufacture
Bosentan	Bosentan tablets 62.5 mg 「JG」	Nihon Generic Co., Ltd
Fidaxomicin	Dafclir tablets 200 mg	Zeria Pharmaceuticals Co., Ltd
Pranlukast	Onon capsules 112.5 mg	Ono Pharmaceuticals Co., Ltd
Rifaximin	Rifxima tablets 200 mg	ASKA Pharmaceuticals Co., Ltd
Celecoxib	Celecox tablet 200 mg	Astellas Pharma Inc
Danazol	BONZOLE tablet 100 mg	Mitsubishi Tanabe Pharma Corporation

The equilibrium solubility values (*S*_*dissolv*_) of bosentan, fidaxomicin, and rifaximin were measured in this study. The *S*_*dissolv*_ values of the other drugs are taken from the literature. The initial pH values of FaSSIF and FeSSIF were aligned to pH 6.5. After incubation for 72 h, the final pH value was pH 6.50 ± 0.01 in all cases. In the cases of bosentan and fidaxomicin, the solid form remained the same before and after the solubility measurements (SI Figure S1). In the case of rifaximin, the original anhydrate form transformed to a hydrate form after the solubility measurement. In all cases, *S*_*dissolv*_ was higher in FeSSIF than in FaSSIF, especially for pranlukast (FeSSIF/FaSSIF = 9.1-fold) (Table I).

### Rate-Limiting Step of Oral Drug Absorption and Food Effect Prediction

Based on the physicochemical properties, the gastric dissolution process was considered negligible for the oral absorption of the model drugs. Therefore, Eq. (1) can be used to calculate *Fa*. Previously, FaRLS of pranlukast, celecoxib, and danazol have been diagnosed to be SL-E, SL-U, and SL-U, respectively [74]. In the present study, the FaRLSs of the other model drugs were diagnosed. The dissolution rate constant (*k*_*diss*_) was calculated from the initial slope of the dissolution curve in the μFLUX experiments and converted to *Dn* by Eq. (2). *Pn* was calculated using the Caco-2 *P*_*app*_ assuming *P*_*ep,u*_ = *P*_*app*_ (< 1 × 10^–6^ cm/s) (Eqs. (3), (7), (8), *P*_*UWL*_ <  < *P*_*ep*_*’*). The dose number (*Do*) was calculated using *S*_*dissolv*_ in FaSSIF and FeSSIF (Eq. (4)). In all cases, *Dn* >  > *Pn*/*Do, Do* > *1, and P*_*ep’*_ <  < *P*_*UWL*_*.* Therefore, the oral absorption of these drugs was diagnosed to be SL-E (Supplemental Information Table S3).

As mentioned in the theory section, for SL-E drugs, the food effect in humans (fed/fasted *Fa* ratio) and the *J*_*μFLUX*_ ratio in μFLUX are predicted to be 1.2 and 1.0, respectively, irrespective of bile micelle solubilization. For SL-U drugs, the food effect is predicted to be positive [26]. Previously, the GUT framework predicted the food effect to be 2.3 (celecoxib) and 3.5 (danazol) [74].

Eq. (17) has often been used in PBBM using commercial software, such as GastroPlus and simCYP. In this case, as mentioned in the theory section, *Fa*_*fed*_/*Fa*_*fasted*_ would be approximately predicted to be the fed/fasted *S*_*dissolv*_ ratio for both the SL-E and SL-U drugs.

### μFLUX Experiments

**Fig. 4:**
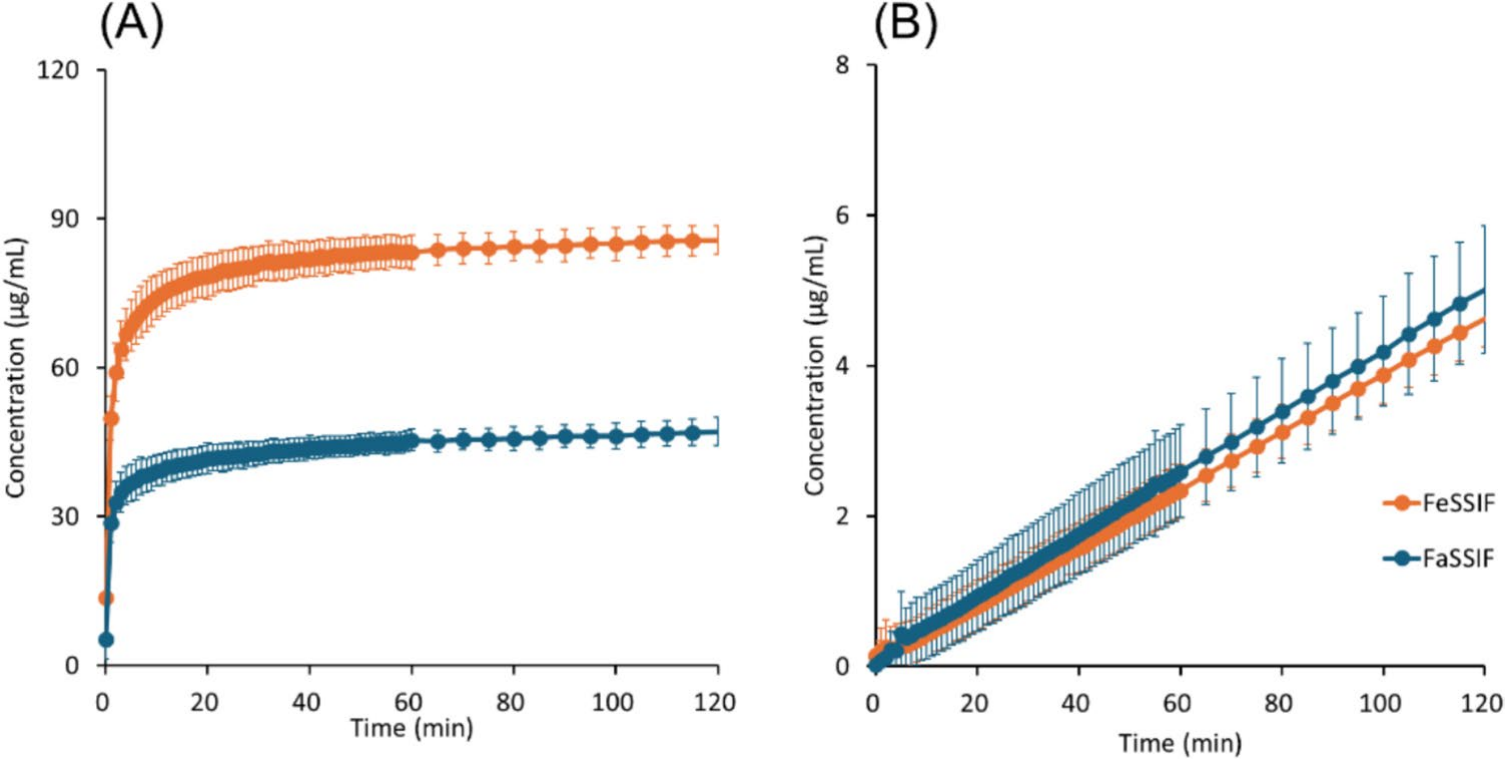
Drug concentration–time profiles in μFLUX for bosentan. (**A**) Dissolved drug concentrations in the donor solution, (**B**) drug concentrations in the acceptor solution. Mean ± S.D., *N* = 3.

**Fig. 5:**
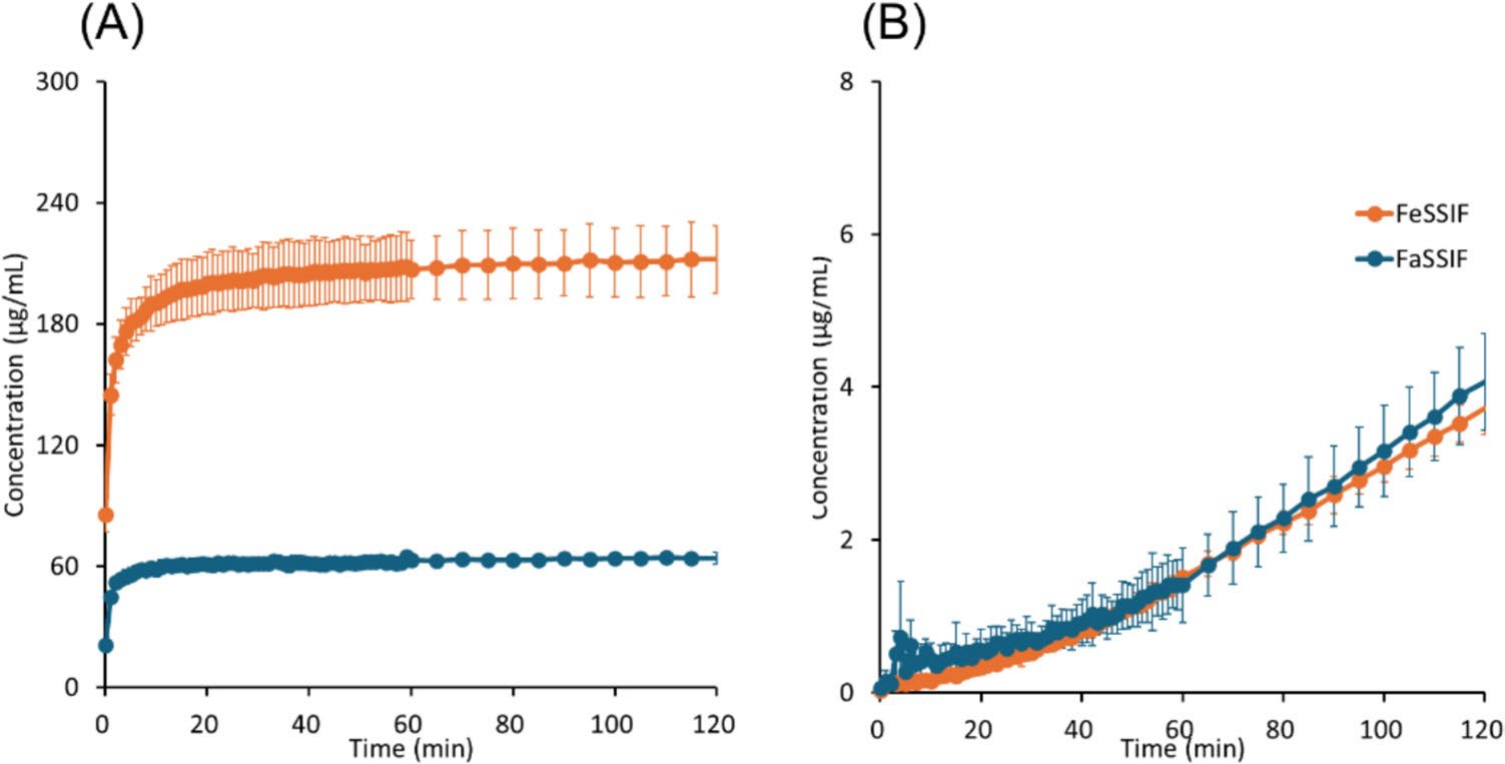
Drug concentration–time profiles in μFLUX for fidaxomicin. (**A**) Dissolved drug concentrations in the donor solution, (**B**) drug concentrations in the acceptor solution. Mean ± S.D., *N* = 3.

**Fig. 6:**
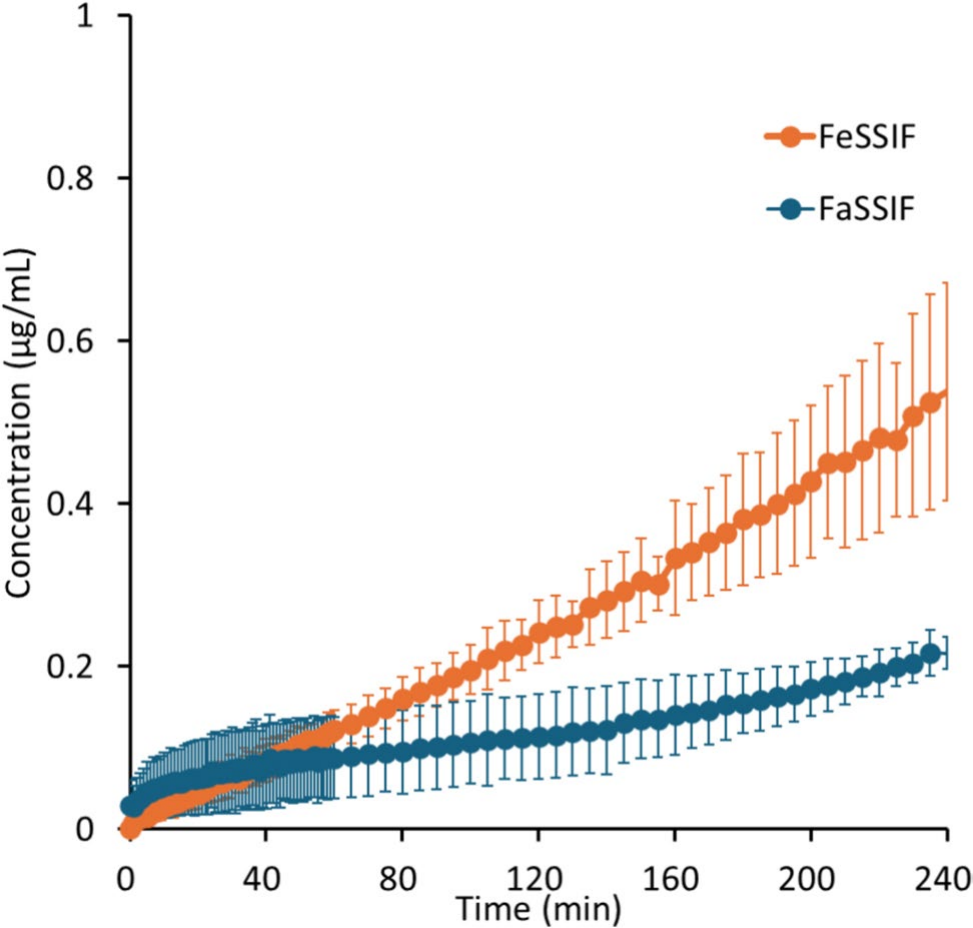
Drug concentration–time profiles in μFLUX for pranlukast. The drug concentration–time profile in the donor solution is not shown because it could not be measured by the *in situ* UV probe due to a large turbidity. Mean ± S.D., *N* = 3.

**Fig. 7:**
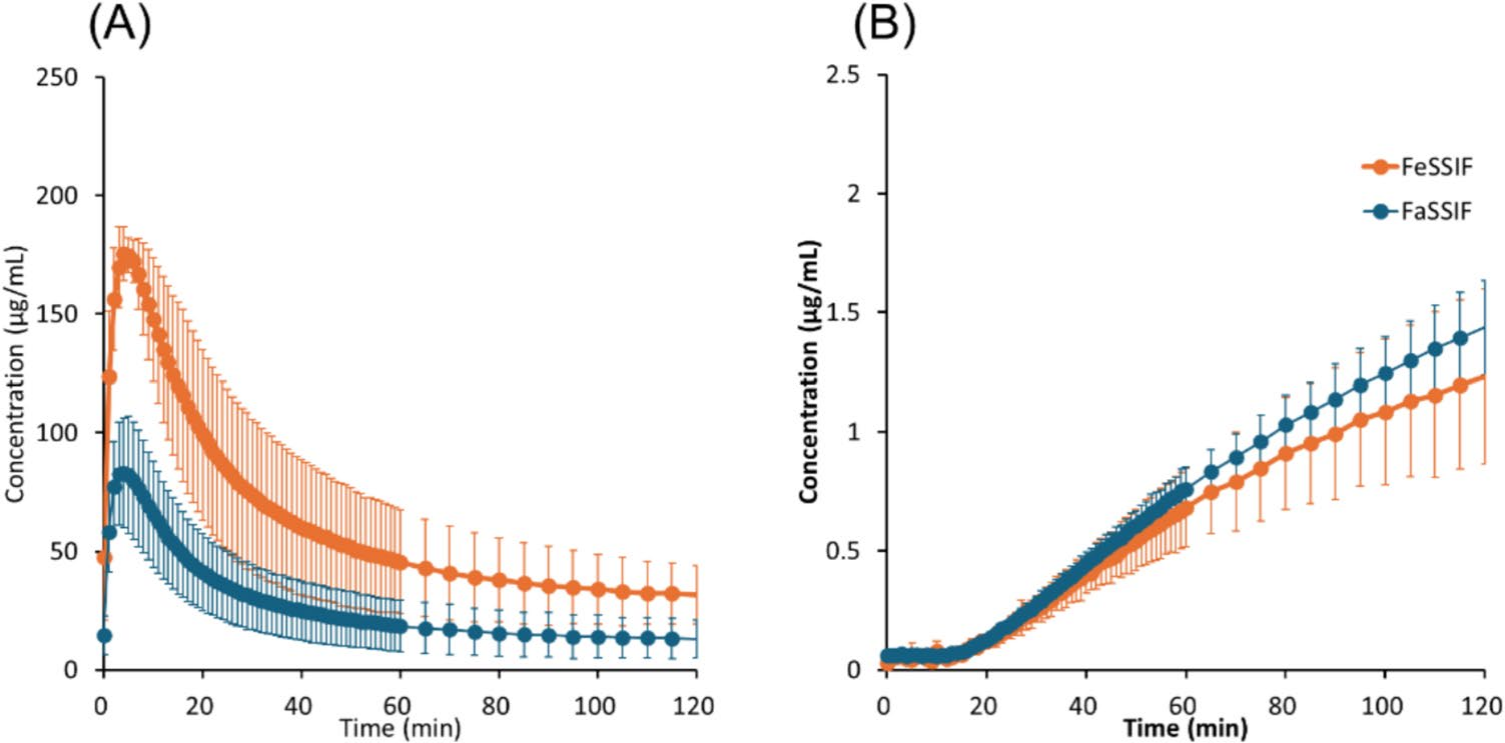
Drug concentration–time profiles in μFLUX for rifaximin. (**A**) Dissolved drug concentrations in the donor solution, (**B**) drug concentrations in the acceptor solution. Mean ± S.D., *N* = 3.

**Fig. 8:**
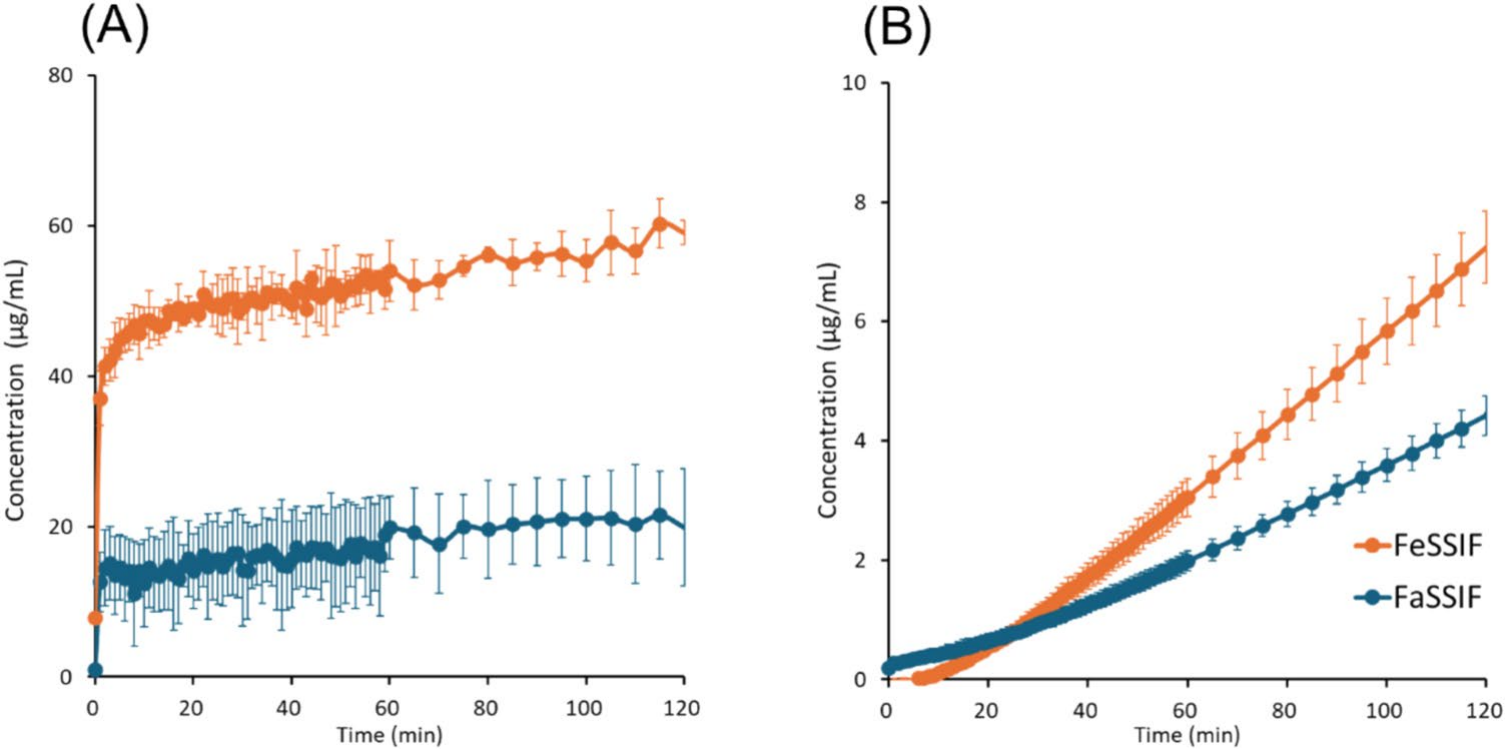
Drug concentration–time profiles in μFLUX for celecoxib. (**A**) Dissolved drug concentrations in the donor solution, (**B**) drug concentrations in the acceptor solution. Mean ± S.D., *N* = 3.

**Fig. 9:**
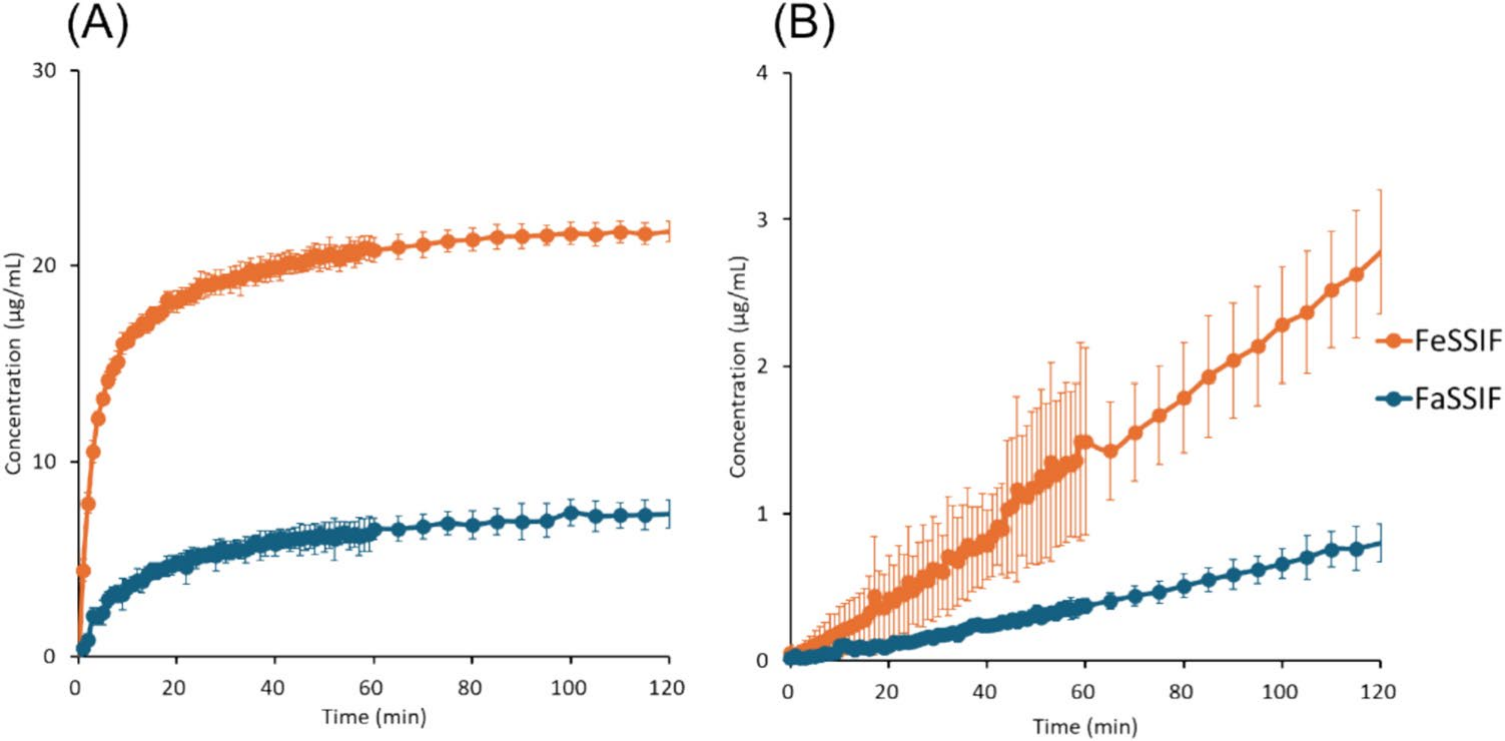
Drug concentration–time profiles in μFLUX for danazol. (**A**) Dissolved drug concentrations in the donor solution, (**B**) drug concentrations in the acceptor solution. Mean ± S.D., *N* = 3.

The results of μFLUX experiments are shown in Figs. 4, 5, 6, 7, 8 to 9 and summarized in Table IV. The dose amount was set to 1/10 of the clinical dose to approximately align the dose/fluid volume ratio between humans and μFLUX [75, 76]. The *in situ* UV measurement of a drug concentration dissolved in the donor solution (*C*_*D*_) can be interfered with by the background scattering from the undissolved drug and insoluble excipients. Especially in the case of pranlukast, *C*_*D*_ could not be measured due to a large turbidity. Therefore, the final drug concentration in the donor solution (*C*_*D,final*_) was also measured after filtration. In the case of pranlukast, the incubation time was prolonged from 2 to 4 h because the drug concentration in the acceptor solution (*C*_*A*_) was low and close to the detection limit by the UV probe.

**Table IV:** μFLUX Results (mean ± S.D., N = 3)

Drug	Donor media ^a^	*C*_D,final_^b^ (μg/mL)	*J*_*μFLUX*_^c^ (10–^3^ μg/cm^2^/s)	*P*_eff,μFLUX_^d^ (10^–5^ cm/s)	*f*_*u*_^*e*^	*P*_*ep,u,μFLUX*_^*f*^ (10^–5^ cm/s)	*P*_*UWL*_^*g*^ (10^–5^ cm/s)
SL-E							
Bosentan	FaSSIF	49 ± 0	6.7 ± 0.8	14 ± 2	0.73	18 ± 2	26
	FeSSIF	95 ± 0	6.1 ± 0.2	6.4 ± 0.2	0.34	19 ± 1	16
Fidaxomicin	FaSSIF	72 ± 0	7.4 ± 0.7	10 ± 1	0.60	17 ± 2	16
	FeSSIF	220 ± 0^ h^	6.0 ± 0.7	2.7 ± 0.3	0.26	10 ± 1	11
Pranlukast	FaSSIF	56 ± 1	0.20 ± 0.05	0.37 ± 0.09	0.038	10 ± 2	2.1
	FeSSIF	1100 ± 0^ h^	0.47 ± 0.16	0.043 ± 0.015	0.0041	10 ± 4	6.5
Rifaximin	FaSSIF	11 ± 1	1.6 ± 0.3	15 ± 2	0.53	28 ± 4	16
	FeSSIF	36 ± 14	1.3 ± 0.5	3.4 ± 0.2	0.15	23 ± 1	10
SL-U							
Celecoxib	FaSSIF	37 ± 1	6.7 ± 0.4	18 ± 2	0.069	-	3.6
	FeSSIF	62 ± 1	11 ± 1	18 ± 1	0.031	-	7.5
Danazol	FaSSIF	7.4 ± 0.3	1.2 ± 0.2	16 ± 3	0.011	-	1.3
	FeSSIF	24 ± 1	4.0 ± 0.2	16 ± 1	0.0043	-	6.6

a pH 6.5 for both FaSSIF and FeSSIF. b Measured after filtration. c The average of 90 to 120 min except for pranlukast (210 to 240 min). d Calculated based on *C*_*D,final*_. e Calculated by Eq. (9). f For SL-U, *P*_*ep,u*_ cannot be calculated because the permeation is limited by UWL. g Predicted by Eq. (10). h Two significant digits

In the donor solution, *C*_*D, final*_ was markedly greater in FeSSIF than in FaSSIF in all cases, especially for pranlukast (20-fold). *C*_*D*_ reached a plateau after 20 min for bosentan, fidaximicin, celecoxib, and danazol, suggesting that the absorption became solubility-limited. *C*_*D,final*_ was close to the equilibrium solubility (*S*_*dissolv*_), except for rifaximin. In the case of rifaximin, *C*_*D*_ showed a supersaturation-precipitation profile. The maximum *C*_*D*_ (*C*_*max*_) was about 90 μg/mL in FaSSIF and 170 μg/mL in FeSSIF. The time to *C*_*max*_ (*T*_*max*_) was about 10 min. After *T*_*max*_, *C*_*D*_ gradually decreased. However, *C*_*D,final*_ was still higher than *S*_*dissolv*_ after 2 h.

In the acceptor solution, the *C*_*A*_-time profiles and *J*_*μFLUX*_ were almost identical between the fasted and fed conditions for the SL-E drugs (the ratio of *J*_*μFLUX*_ ≈ 1.0), except for pranlukast (the ratio of *J*_*μFLUX*_ = 2.4). In the case of bosentan and fidaxomicin, the *C*_*A*_-time profiles reached a steady state. In the case of pranlukast, the slope of *C*_*A*_-time profiles continued to increase at 4 h. In the case of rifaximin, the slope of *C*_*A*_-time profiles continued to decrease at 2 h. In the case of the SL-U drugs, the *C*_*A*_-time profiles and *J*_*μFLUX*_ were higher in the fed condition than in the fasted condition.

In the case of the SL-E drugs, *P*_*eff,μFLUX*_ was less than *P*_*UWL*_ predicted by Eq. (10). *P*_*eff,μFLUX*_ in the fed condition was lower than that in the fasted condition (cf. *P*_*eff,μFLUX*_ was calculated based on the sum of bile micelle-bound and unbound drug concentrations in the donor solution (*C*_*D,final*_) (Eq. (20))). In the case of the SL-U drugs, *P*_*eff,μFLUX*_ was greater than *P*_*UWL*_ predicted by Eq. (10). *P*_*eff,μFLUX*_ in the fed condition was similar to that in the fasted condition.

For the SL-E drugs, the membrane permeability of unbound drug molecules was calculated (*P*_*ep,u,μFLUX*_ = *P*_*eff,μFLUX*_/*f*_*u*_). *P*_*ep,u,μFLUX*_ in the fed condition was similar to that in the fasted condition except for fidaxomicin (about 40% less in the fed condition).

### Comparison With Clinical Food Effect

**Fig. 10:**
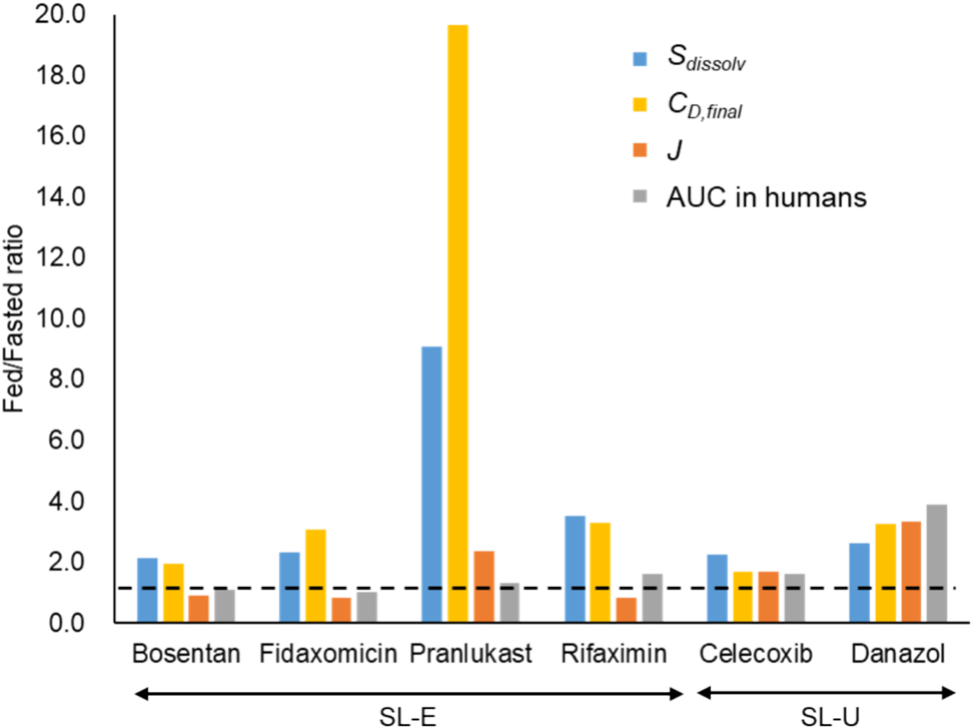
Comparison of the fed/fasted ratios of *S*_*dissolv*_, *C*_*D,final*_, *J*_*μFLUX*_, and clinical AUC. The dotted line is the predicted *Fa* ratio for SL-E (ratio = 1.2). The fed/fasted *S*_*dissolv*_ ratio corresponds to *Fa*_*fed*_/*Fa*_*fasted*_ predicted by Eq. (18).

In the case of the SL-E drugs, the theoretical food effect prediction, that is, the fed/fasted *Fa* ratio = 1.2, is in good agreement with the clinical food effect (the fed/fasted AUC ratio (AUCr)), assuming no food effect on first pass intestinal and hepatic metabolisms (Table V, Fig. 10). In contrast, the *S*_*dissolv*_ and *C*_*D,final*_ ratios (FeSSIF/FaSSIF) were significantly greater than AUCr.

**Table V:** Prediction of Food Effect By Bile Micelles^a^

Drug	Clinical dose (mg)	Dose number		Ratio (fed/fasted)				Ref
		Fasted	Fed	*S*_*dissolv*_^*b*^	*C*_*D,final*_^*c*^	*J*_*μFLUX*_^*c*^	AUCr ^*d*^	
SL-E								
Bosentan	125	23	9	2.1	1.9	0.91	1.1	[48]
Fidaxomicin	200	20	7	2.3	3.1	0.81	1.0	[49]
Pranlukast	225	20	2	9.1	20	2.4	1.3	[50]
Rifaximin	550	12 × 10^2^	2.9 × 10^2^	3.5	3.3	0.81	1.6	[51]
SL-U								
Celecoxib	400	67	25	2.2	1.7	1.7	1.6	[77]
Danazol	100	43	14	2.6	3.2	3.3	3.9	[78]

a For SL-E drugs, the food effect by bile micelles is predicted to be 1.2 regardless of bile micelle solubilization. b This corresponds to *Fa*_*fed*_/*Fa*_*fasted*_ predicted by Eq. (18). c The μFLUX dose was 1/10 of the clinical dose. d Clinical AUC ratios in the fed/fasted conditions

When using Eq. (17) that ignores the free drug theory and UWL for *P*_*eff*_ calculation, *Fa*_*fed*_/*Fa*_*fasted*_ is predicted to be about the same as the fed/fasted *S*_*dissolv*_ ratio (Eq. (18)), significantly overestimating the food effect for the SL-E drugs.

The *J*_*μFLUX*_ ratio was more consistent with AUCr than the *S*_*dissolv*_ and *C*_*D,final*_ ratios. In the case of the SL-U drugs, the *S*_*dissolv*_, *C*_*D,final*_, and *J*_*μFLUX*_ ratios were approximately close to AUCr.

## Discussion

### Theoretical Food Effect Prediction

In the case of the SL-E drugs, despite the equilibrium solubility of the model drugs being markedly increased in FeSSIF compared to FaSSIF, no or a slightly positive food effect had been observed in humans (Table V, Fig. 10). Therefore, the food effect was appropriately predicted by Eq.  (13) for the four SL-E drugs [26]. In the case of the SL-U drugs, the increase in solubility in the fed state condition translated to the positive food effect on *Fa*, in good agreement with previous publications [26, 45, 79].

### μFLUX Data

To confirm the above-mentioned mechanism, the effect of bile micelles on the flux value was further evaluated by the μFLUX experiments. The dissolved drug concentration in the donor fluid (*C*_*D*_) was significantly increased in FeSSIF compared to FaSSIF in all cases. However, in the case of the SL-E drugs, *J*_*μFLUX*_ was little or less affected. *P*_*eff,μFLUX*_ was significantly reduced in the fed condition, whereas *P*_*ep,u,μFLUX*_ was little or less affected. These results are in good agreement with the theoretical prediction for SL-E drugs.

In the case of pranlukast, the *J*_*μFLUX*_ ratio was 2.4-fold. When compared to the *CD,*_*final*_ ratio (20-fold), this *J*_*μFLUX*_ ratio is much smaller. This is in line with the free drug theory. However, the *J*_*μFLUX*_ ratio was still higher than the theoretically predicted value (1.0). One hypothesis is that, since *J*_*μFLUX*_ did not reach a steady state at 4 h, the *J*_*μFLUX*_ ratio may become close to 1.0 at a longer time point. Another hypothesis is that a small portion of bile micelle-bound drugs might have been directly transferred to the membrane. However, in the dissolution/permeation system using Caco-2, this phenomenon was not observed [14]. It is important to note that pranlukast represents an extreme case of exceptionally high bile micelle solubilization. In FeSSIF, pranlukast was 99.6% bound to bile micelles (*f*_*u*_ = 0.004). Therefore, if only 0.6% of bile micelle-bound drug molecules were directly transferred to the membrane, *J*_*μFLUX*_ would increase 2.4-fold.

In the case of rifaximin, significant supersaturation was observed in the early time points. This supersaturated concentration increased the flux value (the slope of the *C*_*A*_-time profile). This is in good agreement with previous findings for amorphous solid dispersion [80, 81]. There is little difference in *J*_*μFLUX*_ between FaSSIF and FeSSIF, suggesting that the unbound drug concentrations in FaSSIF and FeSSIF were the same in the supersaturated state.

In the case of the SL-U drugs, the increase in *C*_*D*_ translated to an increase in *J*_*μFLUX*_, in good agreement with previous publications [14, 35, 36]. *P*_*eff,μFLUX*_ was greater than *P*_*UWL*_, suggesting the contribution of the particle drifting effect in μFLUX [82–86]. This point is currently under investigation.

### Solubility-Permeability Trade-Off

The interplay between solubility and permeability discussed above is often referred to as “solubility-permeability trade-off” for solubilization enhancers such as surfactants and cyclodextrins [87–90]. Solubility-permeability trade-off is often clearly observed in *in vitro* dissolution-permeation flux experiments. However, it is not clearly observed *in vivo*, or not at all. A recent study provided an example of an *in vitro* dissolution-permeation flux experiment overestimating the solubility-permeability trade-off [91]. This may be due to the difference in the membrane surface area between *in vivo* and *in vitro*. Due to the significant expansion of the surface area by villi (tenfold in humans), drug permeation tends to be UWL-limited in the *in vivo* small intestine. On the other hand, because a planar membrane is used, drug permeation can become EPM limited in the *in vitro* systems for some of these cases. The use of a high stirring speed (150 rpm) further enhances this tendency because it reduces the thickness of UWL and increases *P*_*UWL*_. To understand the differences in solubility-permeability trade-off between *in vivo* and *in vitro* systems, these variations in UWL thickness and the available membrane surface area should be considered.

### Comparison of the Biopharmaceutics Classification System and FaRLS

The biopharmaceutics classification system (BCS) has been used for empirical prediction of the food effect [31, 92]. BCS categorizes drugs into 4 classes: (I) high solubility/high permeability, (II) low solubility/high permeability, (III) high solubility/low permeability, and (IV) low solubility/low permeability. BCS I to IV roughly corresponds to PL-U, SL-U, PL-E, and SL-E, respectively (DRL is outside of BCS (discussed below)). BCS II and BCS III are empirically predicted to undergo the positive and negative food effects, respectively. However, BCS IV is not predictable [31, 32, 92].

In contrast, FaRLS mechanistically predicts the food effect on *Fa*. For SL-E drugs, FaRLS predicts the food effect to be little or slightly positive (1.2). Although BCS IV roughly corresponds to SL-E, the rationale for the permeability criterion differs between BCS and FaRLS. In BCS, the high/low permeability criterion is *Fa* = 0.85 or 0.90, corresponding to about *Pn* = 2. This criterion might have been derived from the bioequivalence criteria in regulatory guidance [37]. On the other hand, in FaRLS, the permeability criterion is based on the rate-limiting step being EPM or UWL permeation, which can be diagnosed using the *P*_*eff*_ equation (Eq. (8), Table I) (for many SL-U drugs, *Pn* > 4) [45, 63].

In addition to SL-E drugs, in the case of SL-U drugs (≈ BCS II), FaRLS predicts a positive food effect because both bile micelle-bound and unbound drug molecules can permeate UWL (Fig. 1, Table I) [26]. In the case of PL-E drugs (≈ BCS III), FaRLS predicts a negative food effect because only unbound (free) drug molecules can permeate EPM [11–28, 41]. In the case of PL-U drugs, FaRLS predicts no food effect because *Fa* approaches 1 in both the fasted and fed states (Eq. (1)). In the case of DRL drugs, an increase in *S*_*dissolv*_ by bile micelles would enhance the dissolution rate, resulting in a positive food effect [9, 42, 93]. Taken together, FaRLS provides a consistent mechanistic basis for predicting the food effect on *Fa*.

BCS II has often been mistaken for DRL since the original BCS article was published in 1995 [94]. *Do* and *Pn* define BCS*,* but not *Dn*. For this reason, in the BCS-based biowaiver scheme, *Dn* is evaluated separately from BCS using dissolution tests [37, 95]. In most cases of BCS II drugs, the oral absorption becomes SL-U [63].

### Suggestions For Physiologically Based Biopharmaceutics Modeling

PBBM is believed to be capable of accurately and quantitatively predicting the effect of food on drug absorption [96–100]. However, despite plenty of evidence that bile micelle partitioning reduces *P*_*eff*_ [11–25, 41], this mechanism is largely ignored in most PBBM studies [96]. Rather, a simple empirical correlation (Eq. (17)) has often been used for PBBM, for example, using GastroPlus and simCYP. The use of Eq. (17) implicitly assumes that *P*_*eff*_ is the same in the fasted and fed state. In this case, *Fa*_*fed*_/*Fa*_*fasted*_ may be predicted to be about the same as the fed/fasted *S*_*dissolv*_ ratio (Eq. (18)). As shown in Fig. 10, this significantly overestimated the food effect for the SL-E drugs. In addition, Eq. (17) cannot predict the negative food effect by bile micelles for PL-E drugs [24, 43, 44]. To predict the food effect by bile micelles consistently for PL-E, SL-E, and SL-U drugs, both *f*_*u*_ and UWL need to be simultaneously considered in the theoretical *P*_*eff*_ calculation [40]. Equation (8) and its derivatives have already been implemented in several PBBM programs [24, 74, 101–104].

In most cases, the bottom-up PBBM prediction of a plasma concentration (Cp)-time profile deviates from the clinical data. In such cases, it has become common practice to back-calculate one or more parameters from the clinical Cp-time data on a drug-by-drug basis [97]. This strategy is known as the “(local) middle-out approach”. *P*_*eff*_ is often selected as a target parameter. However, as shown in the present study, *P*_*eff*_ in the fed state is highly likely to be lower than that in the fasted state for SL-E and PL-E drugs [105]. Therefore, the *P*_*eff*_ value back-calculated from the Cp-time data in the fasted state should not be used for predicting the food effect. In addition, the types of food may also affect *P*_*eff*_, for example, high-fat *vs.* low-fat [27]. Finally, but most importantly, *P*_*eff*_ cannot be accurately back-calculated from the Cp-time data after the oral administration of a solid dosage form [106, 107].

In μFLUX, in the case of the SL-E and SL-U drugs, the drug concentration in the acceptor compartment increased almost linearly after a short lag time as *C*_*dissolv*_ reached a plateau and remained constant under a non-sink condition (*C*_*dissolv*_ ≈ *S*_*dissolv*_) (except for rifaximin). In this case, the flux value (*J*) became constant, following the pseudo-zero-order kinetics (*J* = *P*_*eff*_*S*_*dissolv*_). Therefore, it is valid to calculate *Fa* in μFLUX as *Fa* = *Pn*/*Do*. On the other hand, in humans, due to greater permeation clearance, as a drug is absorbed (ex, *Fa* after time *t* > 0.7), *C*_*dissolv*_ can decrease to be less than *S*_*dissolv*_ (*C*_*dissolv*_ < *S*_*dissolv*_). In this case, oral drug absorption after time *t* follows the first-order kinetics under a sink condition (*J* = *P*_*eff*_*C*_*dissolv*_^*1*^ (*J *is proportional to the first power of* C*_*dissolv*_)). Therefore, *Fa* = *Pn*/*Do* is not valid [65]. This point would also complicate the back-calculation of *P*_*eff*_ from the Cp-time profiles in humans in the middle-out approach. When extrapolating the μFLUX data to the Cp-time profiles in humans, the finite absorption time (F.A.T.) concept should be considered [108–110].

### Limitations of This Study

The number of model drugs should be increased in the future to further validate the predictability of FaRLS and μFlux for SL-E drugs. In the literature, several drugs were reported to undergo a positive food effect despite being assigned as BCS IV (≈ SL-E), such as nilotinib HCl, venetoclax, anacetrapib, and etravirine [35, 111–113]. These drugs are highly lipophilic (log*P*_*oct*_ > 5). In this case, *in vitro P*_*app*_ might have been underestimated due to the low recovery or poor solubility, resulting in misclassification of BCS [54, 114–118]. A solubilizer, such as albumin, can be added to the donor and acceptor fluids to avoid these artifacts. In such cases, *P*_*app*_ should be corrected by the (free) unbound fraction for BCS classification [117]. Log*P*_*oct*_ of these drugs suggests that they would show high passive permeability [31, 55].

In the present study, gastric dissolution was neglected. The gastric conditions would be important for the oral drug absorption of a weak base drug and the salt form of an acidic drug [45, 119–121]. The gastric conditions in the fed state are also significantly different from those in the fasted state [5]. In the present study, the effect of dietary fat was not considered. Recently, it was suggested that the fat droplets can drift into the UWL and increase *P*_*eff*_ for SL-U drugs [27]. Lipid digestion can also affect *P*_*eff*_ [122, 123]. In addition, the other GI conditions differ between fasted and fed states. For example, food components would increase macro-viscosity, prolonging the disintegration time [124, 125]. However, micro-viscosity would not be increased, so that the diffusion coefficient would not be affected [126].

The effect of carrier-mediated transport was neglected in this study [127]. The poor intestinal permeability of the model SL-E drugs can be due to efflux transport and/or slow passive permeation. *P*_*ep,u,μFLUX*_　of the model SL-E drugs was significantly higher than *P*_*app*_ in Caco-2, suggesting significant efflux transport in Caco-2. Therefore, a cellular membrane such as Caco-2 could be used for a dissolution-permeation flux experiment [14]. However, the artificial lipid membrane was used in μFlux for practical reasons. Food intake can also affect intestinal and hepatic first-pass metabolism, as well as biliary excretion [128, 129].

## Conclusion

FaRLS predicted the food effect for the SL-E drugs appropriately. The μFLUX experiments confirmed the prediction mechanism. Together with the previous results for SL-U and PL-E, FaRLS and μFLUX are suggested to be useful tools to understand and predict the food effect on oral drug absorption in drug discovery and development.

## Supplementary Information

Below is the link to the electronic supplementary material.

ESM1 (DOCX 246 KB)

## Data Availability

The datasets generated during and/or analysed during the current study are available from the corresponding author on reasonable request.

## Abbreviations

AUC: Area under the plasma concentration–time curve
DRL: Dissolution rate limited
EPM: Epithelial membrane
FaRLS: Fa rate-limiting step
GI: Gastrointestinal
GUT framework: Gastrointestinal unified theoretical framework
PL-E: Epithelial membrane permeability limited
PL-U: Unstirred water layer permeability limited
RLS: Rate-limiting step
SL-E: Solubility—epithelial membrane permeability limited
SL-U: Solubility—unstirred water layer permeability limited
UWL: Unstirred water layer

## Parameter names

*C*_*bm*_: Bile acid concentration
*DF*: Degree of flatness
*Dn*: Dissolution number
*Do*: Dose number
*Dose*: Dose strength
*D*_*bm*_: Diffusion coefficient of bile micelle-bound drug
*D*_*mono*_: Diffusion coefficient of unbound monomer drug
*Fa*: Fraction of a dose absorbed
*f*_*u*_: Unbound fraction
*h*_*UWL*_: Thickness of UWL
*J*_*μ*__*FLUX*_: Flux in μFLUX
*K*_*a*_: Dissociation constant
*k*_*diss*_: Dissolution rate constant
*k*_*perm*_: First-order permeation rate constant
*K*_*bm*_: Bile micelle partition coefficient of unionized species
*MW*: Molecular weight
*P*_*app*_: Apparent permeability
*PE*: Plica expansion factor
*P*_*eff*_: *in vivo* Effective intestinal membrane permeability
*P*_*eff*__*,*__*μ*__*FLUX*_: Effective permeability in μFLUX
*P*_*ep*__*,u*_: Epithelial membrane permeability for unbound (free) drug molecules
*P*_*ep*__*,*__*μ*__*FLUX*_: Artificial membrane permeability for unbound (free) drug molecules
*P*_*ep’*_: Effective epithelial membrane permeability
*Pn*: Permeation number
*P*_*oct*_: Octanol-water partition coefficient
*P*_*UWL*_: Unstirred water layer permeability
*P*_*WC*_: Permeation by water conveyance
*R*_*SI*_: Radius of the small intestine
*S*_*dissolv*_: Dissolved drug concentration in the intestinal fluid
*T*_*SI*_: Small intestinal transit time
*VE*: Villi expansion factor
*V*_*SI*_: Small intestinal fluid volume
