# Supplementary material for: Predicting Food Effect On Oral Drug Absorption For Solubility-Epithelial Membrane Permeation-Limited Cases With Bile Micelle Solubilization
Source: Pharm Res. 2025 Oct 14;42(10):1837–55. doi: 10.1007/s11095-025-03947-8 (PMC12592319; doi:10.1007/s11095-025-03947-8)
Supplement: Supplementary file 1 — (DOCX 246 KB) [file 11095_2025_3947_MOESM1_ESM.docx]

Supplemental information

Predicting food effect on oral drug absorption for solubility-epithelial membrane permeation-limited cases with bile micelle solubilization

Yuji Higashiguchi^1^, Shiori Ishida^1^, Samuel Lee^2^, Balint Sinko^2^, Karl Box^2^, and Kiyohiko Sugano^1*^

1. Molecular Pharmaceutics Lab., College of Pharmaceutical Sciences, Ritsumeikan University, 1-1-1, Noji-higashi, Kusatsu, Shiga 525-8577, Japan

2. Pion Inc. (UK) Ltd. Forest Row Business Park, Station Road, East Sussex, RH18 5DW, United Kingdom

* Corresponding author. Tel.: +81-77-561-2773; E-mail address: suganok@fc.ritsumei.ac.jp (K. Sugano).

1. PXRD data of residual solid in solubility measurement


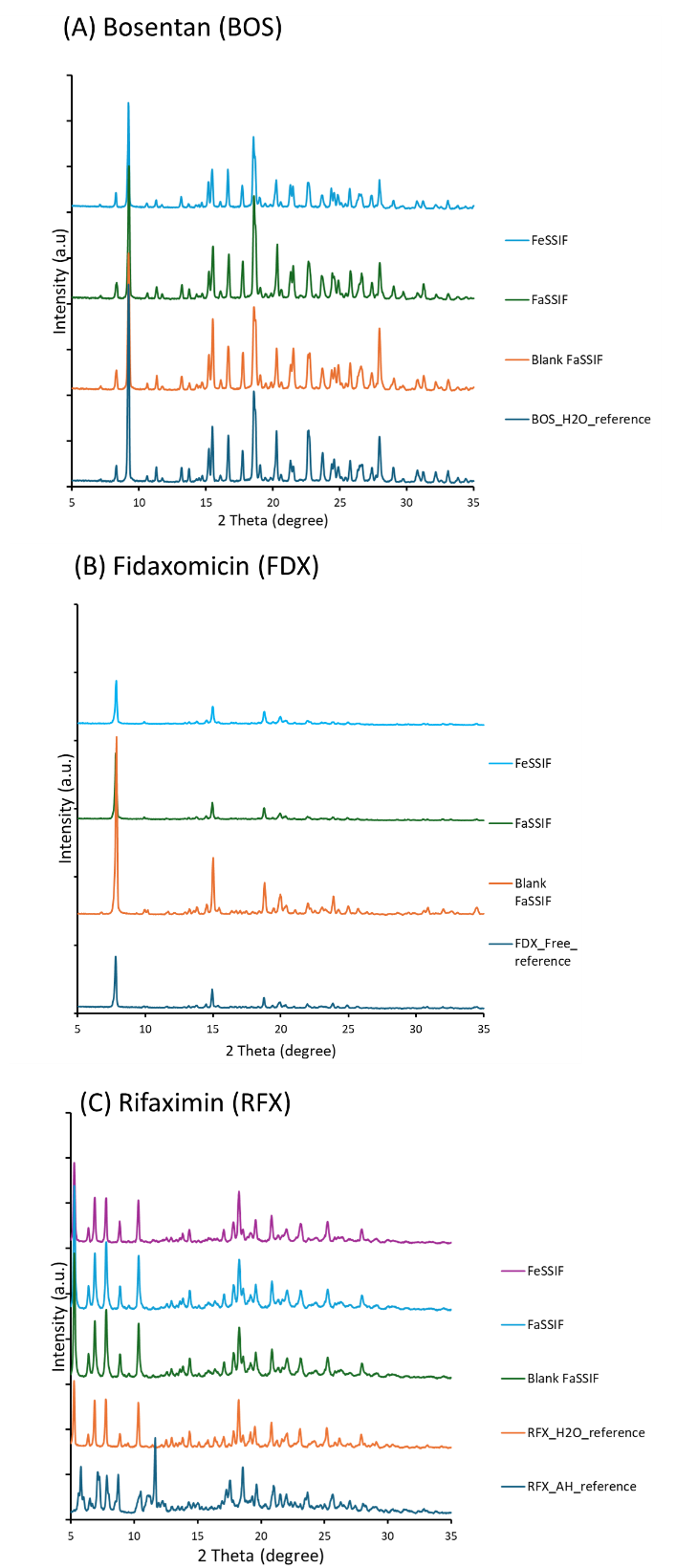


Figure S1 PXRD of residual solid in solubility measurement. Rigaku Ultima IV, Rigaku Corporation, Tokyo, Japan). Data was collected from 5 to 35° (2θ) at a step size of 0.02° and scanning speed of 10 deg/min with Cu Kα radiation generated at 40 mA and 40 kV.

1. Standard curves for solubility and μFLUX measurements

Table S1 Standard curves for solubility measurements

| Drug | UV (nm) | Buffer | Range (μg/mL) | r^2^ |
| --- | --- | --- | --- | --- |
| Bosentan | 276 | Blank FaSSIF | 0.946-30.3 | 0.9993 |
|  |  | FaSSIF | 3.78-30.2 | 0.9995 |
|  |  | FeSSIF | 3.78-60.5 | 0.9993 |
| Fidaxomicin | 316 | Blank FaSSIF | 3.13-100 | 0.9993 |
|  |  | FaSSIF | 3.13-100 | 0.9990 |
|  |  | FeSSIF | 3.13-100 | 0.9963 |
| Rifaximin | 450 | Blank FaSSIF | 1.60-12.8 | 0.9994 |
|  |  | FaSSIF | 1.60-12.8 | 0.9996 |
|  |  | FeSSIF | 1.60-25.6 | 1.0000 |

Table S2 Standard curves for μFLUX measurements

| Drug | UV(nm) | Buffer | Range (μg/mL) | r^2^ |
| --- | --- | --- | --- | --- |
| Bosentan | 276 | FaSSIF (Donor) | 0-70.06 | 1.0000 |
|  |  | FaSSIF (Acceptor) | 0-7.195 | 1.0000 |
|  |  | FeSSIF (Donor) | 0-114.5 | 1.0000 |
|  |  | FeSSIF (Acceptor) | 0-7.195 | 0.9999-1.0000 |
| Fidaxomicin | 316 | FaSSIF (Donor) | 0-65.94 | 0.9988-0.9989 |
|  |  | FaSSIF (Acceptor) | 0-6.107 | 0.9996-0.9999 |
|  |  | FeSSIF (Donor) | 0-158.0 | 0.9990-0.9991 |
|  |  | FeSSIF (Acceptor) | 0-7.611 | 0.9999 |
| Pranlukast | 260 | FaSSIF (Acceptor) | 0-0.4851 | 0.9998-0.9999 |
|  |  | FeSSIF (Acceptor) | 0-0.8079 | 0.9999-1.0000 |
| Rifaximin | 450 | FaSSIF (Donor) | 0-118.3 | 0.9976-0.9990 |
|  |  | FaSSIF (Acceptor) | 0-6.028 | 0.9994-0.9997 |
|  |  | FeSSIF (Donor) | 0-209.5 | 0.9995 |
|  |  | FeSSIF (Acceptor) | 0-6.190 | 0.9991-0.9993 |
| Celecoxib | 280 | FaSSIF (Donor) | 0-59.36 | 0.9998-0.9999 |
|  |  | FaSSIF (Acceptor) | 0.-7.966 | 0.9980-0.9985 |
|  |  | FeSSIF (Donor) | 0-78.95 | 0.9998 |
|  |  | FeSSIF (Acceptor) | 0-15.92 | 0.9998 |
| Danazol | 286 | FaSSIF (Donor) | 0-21.58 | 0.9998 |
|  |  | FaSSIF (Acceptor) | 0-3.017 | 0.9999-1.0000 |
|  |  | FeSSIF (Donor) | 0-70.78 | 1.0000 |
|  |  | FeSSIF (Acceptor) | 0-6.028 | 0.9997-0.9998 |

| Drug | Fed/Fasted | *P_ep,u_*^a^ | *f_u_* | *P_ep_* | *P_ep_*_'_ | *D_mono_*^c^ | *D_eff_*^d^ | *P_UWL_* | *P_eff_* | *k_perm_* | *k_diss_*^b^ | *Pn* | *Dn* | *Do* | FaRLS |
| --- | --- | --- | --- | --- | --- | --- | --- | --- | --- | --- | --- | --- | --- | --- | --- |
|  |  | 10^-6^ cm/s |  | 10^-6^ cm/s | 10^-6^ cm/s | 10^-6^ cm^2^/s | 10^-6^ cm^2^/s | 10^-4^ cm/s | 10^-4^ cm/s | 1/h | 1/h |  |  |  |  |
| Bosentan | Fasted | 1 | 0.73 | 0.73 | 7.3 | 5.7 | 4.3 | 1.7 | 0.21 | 0.17 | 1.695 | 0.63 | 5.9 | 23 | SL-E |
| Bosentan | Fed | 1 | 0.34 | 0.34 | 3.4 | 5.7 | 2.6 | 1.1 | 0.10 | 0.082 | 2.599 | 0.30 | 9.1 | 9.2 | SL-E |
| Fidaxomicin | Fasted | 0.092 | 0.60 | 0.055 | 0.55 | 4.2 | 2.7 | 1.1 | 0.016 | 0.013 | 1.068 | 0.047 | 3.7 | 20 | SL-E |
| Fidaxomicin | Fed | 0.092 | 0.26 | 0.024 | 0.24 | 4.2 | 1.9 | 0.86 | 0.007 | 0.0058 | 2.660 | 0.020 | 9.3 | 7.2 | SL-E |
| Rifaximin | Fasted | 1 | 0.53 | 0.53 | 5.3 | 4.8 | 2.7 | 1.2 | 0.15 | 0.12 | 0.713 | 0.45 | 2.5 | 12×10^2^ | SL-E |
| Rifaximin | Fed | 1 | 0.15 | 0.15 | 1.5 | 4.8 | 1.6 | 0.78 | 0.04 | 0.036 | 1.245 | 0.13 | 4.4 | 2.9×10^2^ | SL-E |

Table S3 FaRLS diagnosis of bosentan, fidaxomicin, and rifaximin [1]

a Assumed to be the same as Caco-2 *P_app_*_._

b Calculated from the initial dissolution rate in μFLUX.

c Calculated from MW (*D_mono_* (cm^2^/s) = 9.9 × 10^-5^ × MW^-0.453^ [2].

d *D_bm_* in FaSSIF was multiplied 3-fold to consider the mucus effect [3,4]

References

1. Sugano K. Fraction of a dose absorbed estimation for structurally diverse low solubility compounds. Int J Pharm. 2011;405.

2. Avdeef A. Absorption and Drug Development: Solubility, Permeability, and Charge State, Second Edition. John Wiley & Sons, Inc.; 2012.

3. Li C-Y, Zimmerman CL, Wiedmann TS. Diffusivity of Bile Salt/Phospholipid Aggregates in Mucin. Pharm Res. 1996;13:535–41.

4. Hanio S, Möllmert S, Möckel C, Choudhury S, Höpfel AI, Zorn T, et al. Bile Is a Selective Elevator for Mucosal Mechanics and Transport. Mol Pharm. 2023;20:6151–61.
